# Genetic Polymorphisms in the Vitamin D Pathway and Non-small Cell Lung Cancer Survival

**DOI:** 10.1007/s12253-019-00702-4

**Published:** 2019-10-17

**Authors:** Jinyu Kong, Xiaojie Chen, Jian Wang, Jingxin Li, Fangxiu Xu, Shegan Gao, Herbert Yu, Biyun Qian

**Affiliations:** 1 Henan Key Laboratory of Cancer Epigenetics; Cancer Institute, The First Affiliated Hospital, and College of Clinical Medicine of Henan University of Science and Technology, Luoyang, 471003 China; 2 Department of Cancer Epidemiology and Biostatistics, National Clinical Research Center of Cancer, Key Laboratory of Cancer Prevention and Therapy, Tianjin Medical University Cancer Institute and Hospital, Tianjin, 300060 China; 3 Hongqiao International Institute of Medicine, Shanghai Tongren Hospital and Faculty of Public Health, Shanghai Jiao Tong University School of Medicine, 227 South Chongqing Road, Shanghai, 200025 China; 4 Medical College, Henan University of Science and Technology, Luoyang, 471003 Henan China; 5 Department of Image Diagnoses, The First Affiliated Hospital, and College of Clinical Medicine, Henan University of Science and Technology, Luoyang, 471003 China; 6 Epidemiology Program, University of Hawaii Cancer Center, 701 Ilalo Street, Honolulu, HI 96813 USA

**Keywords:** Non-small cell lung cancer, Vitamin D pathway, Single nucleotide polymorphism, Genetic susceptibility, Prognosis

## Abstract

Various genetic polymorphisms have been linked to lung cancer susceptibility and survival outcomes. Vitamin D (VD) regulates cell proliferation and differentiation, inhibits tumor growth and induces apoptosis. Observations from several previous studies including our own suggest that genetic polymorphisms in the VD pathway may be associated with lung cancer risk. The aim of this study is to assess if genetic polymorphisms in the VD pathway are associated with the prognosis of non-small cell lung cancer (NSCLC). Nine single nucleotide polymorphisms (SNPs) in five genes in the VD pathway were genotyped with the TaqMan assays in 542 patients with primary NSCLC, and the relationships between these SNPs and overall survival were evaluated. We found that SNP rs10741657 in the CYP2R1 gene was associated with the prognosis of NSCLC, especially in elderly patients and not being treated with chemotherapy. Some of the VD pathway-related genetic polymorphisms may influence the prognosis of NSCLC. More research is needed to further confirm the finding and test if VD supplements can be used for NSCLC treatment.

## Introduction

Non-small cell lung cancer (NSCLC) is a major form of malignancies in the lung and a leading cause of cancer deaths in the world [1, 2]. Despite advances in the diagnosis and treatment of NSCLC in recent decades, the prognosis for patients is still poor compared with the other cancers, such as breast, prostate, and colorectal, especially in China, with the 5-year survival rate of less than 20% [3]. This difference is attributed to variations in the biology of lung tumors [4]. Therefore, the identification of novel biomarkers for more precise prognostication in NSCLC patients would be very important .

Vitamin D (VD) has many important biologic functions, including control of cell proliferation, regulation of differentiation, inhibition of tumor growth and induction of apoptosis [5–7]. The protective effects of VD against lung cancer have been suggested [8, 9]. The biologic actions of VD and other molecules which transport and mediate its effects are achieved through a series of biochemical reactions which convert a VD precensors to a bioactive VD. The process is catalyzed by a number of metabolizing enzymes, including 25-hydroxylases (CYP2R1 and CYP27A1) which convert pro-VD to 25-hydroxyvitamin D_3_ [25(OH)D_3_] and 1-alpha-hydroxylase (CYP27B1) which transforms 25(OH)D_3_ to a bioactive VD, 1,25-dihydroxyvitamin D_3_ [1,25(OH)_2_D_3_]. Furthermorer, 25-hydroxyvitamin D-24-hydroxylase (CYP24A1) involves VD degradation, vitamin D-binding protein (GC) facilitates VD transportation, and VD nuclear receptor (VDR) binds to 1,25(OH)_2_D_3_ exerting the biologic actions together with VD. Genes encoding cytochrome p450 enzymes (CYPs) are highly polymorphic, and therefore genetic polymorphisms related to the enzymes metabolising VD are speculated to affect VD activities, which may further influence the development and progression of lung cancer.

Although the relationship between polymorphisms in the VDR gene and lung cancer has been investigated recently [10], few studies have evaluated single nucleotide polymorphisms (SNPs) in the genes involved in the VD metabolism and activity in Chinese populations. Based on the results of a genome-wide association study (GWAS) [11] and analysis of bioinformatics data, our previous research selected several SNPs which are either potentially functional or known as tagSNPs in the CYP2R1, CYP27B1, CYP24A1, GC and VDR genes, and analyzed their genotypes in 1264 case and control subjects to determine their association with NSCLC. Associations of these SNPs with NSCLC risk have been discussed and several SNPs were found to be associated with the disease risk. We [10] and others [12] also found that expression of CYP27B1 and CYP2R1 differed by their genotypes in certain SNPs and the expression was associated with lung cancer survival. No studies have evaluated the possible effects of these SNPs on the prognosis of NSCLC. In this report, we discussed the results of our study which examined the relationships of SNPs involved in the VD pathway genes and the overall survival of NSCLC patients.

## Materials and Methods

### Study Subjects

Subjects in the study were recruited from Tianjin Medical University Cancer Hospital (TMUCH). Patients enrolled in the study were newly diagnosed between January 2006 and May 2011, and their diagnosis was histologically confirmed. Disease information on histological type, tumor size, lymph node metastasis, disease stage and treatments was extracted from patient medical records. Study patients were followed for survival outcomes from the date of diagnose to August 20, 2013, or date of death due to recurrence, metastasis or other complications. Follow-up was achieved through clinical visits and regular telephone contacts. The study was approved by the ethical review committee at TMUCH. The methods were performed in accordance with the relevant guidelines and approved regulations. After signing an informed consent, all the participants in the study underwent a questionnaire-based in-person interview, and provided 10 ml blood samples which were collected in an ethylene diamine tetraacetic acid (EDTA) vacutainer tube. Blood samples were processed immediately after collection. Plasma and buffy coats were separated after centrifugation at 2000 rpm for 20 min, and the specimens were stored in a liquid nitrogen tank until DNA extraction and genotyping.

### SNP Selection and Genotyping

Based on information from the dbSNP database, HapMap and Haploview, we chose five major genes on the VD signaling pathway for our study, including CYP2R1, CYP27B1, CYP24A1, VDR, and GC. From these genes, we first identified tagging SNPs with linkage disequilibrium (LD) greater than 0.8 in the five selected genes plus the regions 100 kb up- and downstream of each gene, and then selected SNPs located in the protein coding and promoter regions from the tagging SNPs with minor allele frequency > 5%. A total of nine SNPs met our selection criteria. Of them, four SNPs are in CYP27B1 (rs3782130, rs4646536, rs703842, rs10877012), two in CYP24A1 (rs6068816, rs4809957), one in VDR (rs11574129), one in GC (rs7041), and one in CYP2R1 (rs10741657). Using the polymerase chain reaction (PCR)-based florescence 5’nuclease assay (TaqMan), we genotyped these genetic polymorphisms in the blood of NSCLC patients. Detailed protocols for these TaqMan assays were described in our previous report [10].

### Statistical Analysis

Log-rank test and Cox proportional hazards regression model were employed to compare differences in overall survival between patients with various SNP genotypes. All statistical analyses were performed using the SPSS version 17.0 (SPSS Inc., Chicago, IL). Survival time was defined as the time interval between the date of NSCLC diagnosis and date of death or last follow-up (August 20, 2013).

## Results

### Patient Data and Clinicopathological Features

The study included 542 NSCLC cases. The average age of these patients was 60 years, ranging from 34 to 80 years. Detailed characteristics of the study population are shown in Table 1. We conducted prospective follow-up on these patients. Up to August 20, 2013, a total of 517 patients completed follow-up, and 25 patients lost to follow-up. The rate of loss to follow-up was 4.61%. A total of 278 patients (51.3%) died at the end of follow-up.

**Table 1:** Clinicopathological characteristics of patients

Variable	*N* = 542	%
Age		
<60	272	50.2
≥60	270	49.8
Gender		
Male	344	63.5
Female	198	36.5
BMI^a^		
<24	254	47.4
≥24	282	52.6
Pathology		
SCC	282	52.0
ADC	260	48.0
Chemotherapy		
No	246	45.4
Yes	296	54.6
Clinical stages		
I + II	346	63.8
III + IV	196	36.2
Tumor size^a^		
≤3	222	41.2
>3	317	58.8
LN metastasis^a^		
No	301	57.6
Yes	222	42.4
Distant metastasis		
No	498	91.9
Yes	44	8.1
Death		
No	264	48.7
Yes	278	51.3

^a^ Due to the missing value exists, the number of cases is less than 542 cases

### Associations of SNPs with Lung Cancer Survival

Results of univariate and multivariate survival analyses on SNP’s associations with lung cancer survival are shown in Table 2. Of the 9 SNPs analyzed, only SNP rs10741657 in CYP2R1 was significantly associated with overall survival. Patients with homozygous variant genotype had over 64% longer median survival time than those with homozygous wild genotype (*P* = 0.033). This association remained significant after age, gender, tumor histology, postoperative chemotherapy and disease stage were adjusted in the analysis, the risk reduction for death was more than 30% (adjusted HR = 0.69, 95%CI: 0.46–0.97).

**Table 2:** Association of SNP on the VD metabolic pathways and NSCLC survival

Genotype	Case	Death	MST (month)	Log-rank P	HR^a^(95%CI)
	*n* = 542	*n* = 278			
CYP27B1(rs3782130)				0.263	
CC	208	107	58.7		1.00
CG	262	129	58.2		0.63(0.22–1.77)
GG	72	42	43.7		1.16(0.33–4.18)
CYP27B1(rs4646536)				0.625	
CC	204	103	63.8		1.00
CT	172	85	56.0		1.42(0.73–2.74)
TT	166	90	52.4		1.43(0.68–3.04)
CYP27B1(rs703842)				0.627	
CC	214	109	63.8		1.00
CT	232	116	56.0		1.27(0.67–3.25)
TT	96	53	57.6		1.16(0.45–2.78)
CYP27B1(rs10877012)				0.695	
TT	213	109	58.7		1.00
TG	239	120	56.0		1.28(0.69–1.97)
GG	89	48	51.5		1.39(0.38–1.85)
CYP24A1(rs6068816)				0.072	
CC	191	89	68.2		1.00
CT	285	161	48.1		1.13(0.86–1.49)
TT	66	28	77.4^b^		0.76(0.49–1.19)
CYP24A1(rs4809957)				0.790	
GG	210	103	63.8		1.00
GA	279	149	55.3		0.97(0.74–1.26)
AA	53	26	50.9		0.92(0.58–1.45)
VDR(rs11574129)				0.050	
TT	301	164	52.2		1.00
TC	231	107	75.2		0.85(0.66–1.09)
CC	10	7	17.5		1.44(0.66–3.11)
GC(rs7041)				0.693	
TT	297	157	52.7		1.00
TG	213	104	65.2		0.82(0.64–1.07)
GG	32	17	43.6		1.13(0.67–1.92)
CYP2R1(rs10741657)				**0.033**	
GG	221	125	50.9		1.00
GA	249	124	58.9		0.79(0.61–1.03)
AA	71	28	79.5^b^		**0.69(0.46–0.97)**

^a^Adjusted by gender, age, pathological diagnosis, postoperative chemotherapy, clinical stages; ^b^ Expression with an average survival time when the median survival cannot be calculated; Bold text highlights statistically significant findings

### Subgroup Analysis of SNP rs10741657 and Others

**Fig. 1:**
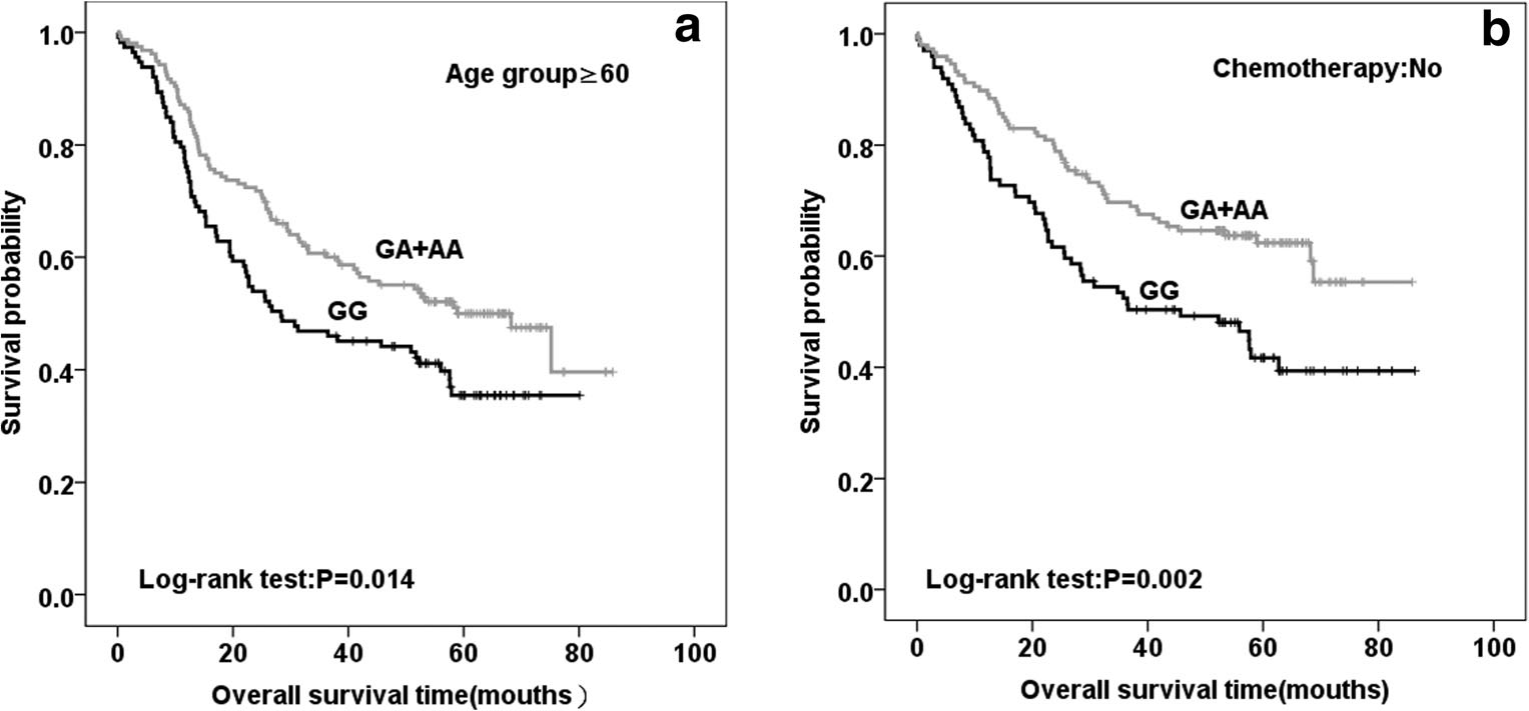
Kaplan–Meier plot of overall survival curves according to CYP2R1 rs10741657G > A genotype in older patients (**a**), and patients without postoperative chemotherapy(**b**)

To control for potential confounding effects, we performed additional survival analyses in subgroups of patients stratified by gender, age, histological type, disease stage and chemotherapy (Table 3). The subgroup analyses showed that the improved survival time in association with the variant genotype of rs10741657, AA or GA, mainly occurred to older patients (Fig. 1a) or without postoperative chemotherapy (Fig. 1b). Adjusted by various factors, the protective effect of genotype ‘AA’ or ‘GA’ for NSCLC patients still showed in the older or without postoperative chemotherapy. Their adjusted HRs were 0.71 (95%CI: 0.51–0.99) for older patients, and 0.65 (95%CI: 0.45–0.95) for those without chemotherapy, respectively.

**Table 3:** The stratified analysis of CYP2R1 rs10741657 and prognosis of NSCLC

Variable	Case/Death		MST (month)		Log-rank P	HR^a^(95%CI)
	GG	GA + AA	GG	GA + AA		
Gender						
Male	139/82	204/99	50.5	68.2	0.064	0.79(0.59–1.06)
Female	82/43	116/53	50.9	68.7	0.334	0.77(0.51–1.15)
Age						
<60	108/55	164/75	62.7	68.7	0.672	0.91(0.64–1.29)
≥60	113/70	156/77	28.3	68.2	**0.014**	**0.71(0.51–0.99)**
Pathology						
SQCC	117/67	165/85	51.7	58.9	0.279	0.83(0.60–1.14)
ADC	104/58	155/67	49.1	–	0.054	0.74(0.52–1.06)
Stage						
I + II	137/57	209/69	–	–	0.131	0.77(0.54–1.09)
III + IV	84/68	111/83	22.7	27.8	0.181	0.82(0.60–1.14)
Chemotherapy						
No	99/56	147/55	45.7	–	**0.002**	**0.65(0.45–0.95)**
Yes	122/69	173/97	51.7	46.4	0.997	0.94(0.69–1.28)

^a^Adjusted by age, gender, pathological diagnosis, clinical stage and postoperative chemotherapy; Bold text highlights statistically significant findings

## Discussion

The development of lung cancer is known to be related to both environmental and genetic factors, SNPs play an important role in genetic susceptibility. Previously, we compared certain SNP genotypes in the VD pathway between NSCLC cases and controls, and found a few associated with lung cancer risk [10]. In this report, we analyzed the association of the VD related SNPs with NSCLC prognosis and found that CYP2R1 rs10741657 was associated with the overall survival of NSCLC.

SNP rs10741657 is located in the promoter region of the CYP2R1 gene. A study by Slater, N.A. et al. [13] showed that non-carriers of rs10741657 risk alleles (CG) had higher levels of 25(OH)D. Ramos-Lopez et al. [12] found a significant association between the genotype of rs10741657 and serum 25(OH)D concentrations in 203 German diabetic patients. Two genome-wide association studies (GWAS) on genetic determinants of serum of vitamin D published in 2010 [14, 15] and found that rs10741657 was significantly associated with 25(OH)D concentrations in 30,000 subjects of European descent from 15 cohorts or in 496 unrelated healthy Caucasian subjects. Lasky-Su et al. [16] conducted a combined analysis of 1164 subjects from two cohorts of Caucasian and Costa Rica asthmatic children, and found that rs10741657 was significantly associated with 25(OH)D concentrations.

Ramos-Lopez etc. [12] studied the correlation between 25(OH)D_3_ and CYP2R1 mRNA with the genotypes of rs10741657, and reported that subjects carrying the ‘GG’ or ‘GA’ genotype of rs10741657 possessed, on average, lower levels of 25(OH)D_3_ compared to those with the ‘AA’ genotype. Others found that rs10741657 was related to pancreatic cancer and glioma pathogenesis [17, 18]. In our study, we found that CYP2R1 rs10741657 was related to the prognosis of lung cancer, and the ‘AA’ genotype was associated with reduced risk of NSCLC death. We also noticed that rs10741657 ‘AA’ alleles was the protective effect of CYP2R1 more evident in elderly patients without postoperative chemotherapy.

The CYP2R1 gene is located on chromosome 11p15.2, and spans about 15.5 kb with 5 exons, CYP2R1 is the key enzyme that converts vitamin D precusor to 25(OH)D in the liver [19]. In the patient with low circulating levels of 25(OH)D_3_ and symptoms of vitamin D deficiency, a study found a transition mutation in exon 2 of the CYP2R1 gene was found, which leads to the substitution of Proline Leucine at amino acid 99 in the CYP2R1 protein and abolishes enzymatic activity of vitamin D 25-hydroxylase, indicating that genetic variation in this gene can affect 25(OH)D synthesis [20].

Research on lung cancer risk and disease progression is often related. The aim of this study is to examine the relationship between the VD pathway-related SNPs and the prognosis of NSCLC. In our previous study we discussed the association between genetic polymorphism and lung cancer risk [10]. Although there have been a lot of studies investigating VD association with lung cancer prognosis, it is the first study to assess the genetypes of various VD-related genes is association with NSCLC prognosis.

Recent studies suggest a possible association between lung cancer development and VD concentration. In 2007, Mohr et al. [21] studied the incidence of lung cancer in 111 countries, and found that ultraviolet radiation (UV) exposure was associated with the incidence of lung cancer. Large survival studies from Norway and England showed that lung cancer patients diagnosed in summer or autumn had longer survival than those diagnosed in winter, suggesting that UV exposure maybe related to lung cancer outcome [22, 23]. Zhou et al. [24] studied 456 NSCLC patients in relation to season of surgery and VD intake. The study found that patients operated in summer and with high VD intake had better 5-year survival and longer overall survival than those operated in winter and with low VD intake. The 5 year survival rates were 56% versus 23%, respectively. Another study in which 447 early stage NSCLC patients were analyzed for their serum 25 (OH) D, found that serum levels of 25 (OH) D and VD intake were association with the overall survival of NSCLC patients with stage IB-IIB disease. Levels of 1,25(OH)_2_D_3_ were positively correlated with overall survival time. Patients with 1,25(OH)_2_D_3_ > 21.6 ng/ml had 26% increase in survival compared to those that 1,25(OH)_2_D_3_ < 10.2 ng/ml [25]. Tretli et al. [26] also confirmed that high serum 25 (OH) D levels was associated with good prognosis of lung cancer.

Genetic polymorphisms have been suspected to partially drive host susceptibility to lung cancer, as well as variations in therapeutic response and prognosis. Our study found a genetic polymorphism in the VD pathway associated with NSCLC prognosis. This finding provides evidence for VD’s link the tumor progression and suggests the possibility that VD may have the therapeutic potential. However, our observation needs further confirmation from independent studies. The prognosis of lung cancer is influenced by many factors, such as histological type, disease state, age at diagnosis, smoking status, sensitivity to chemotherapy drugs or radiation. Genetic polymorphisms may affect some of these factors. Our findings of gene polymorphisms in the VD pathway in relation to NSCLC prognosis is an example of such influences. Studies are also needed to elucidate the mechanisms of CYP2R1 rs10741657 in relation to the prognosis of NSCLC. Through additional studies, we will understand better the role of VD in lung cancer in development and progression.

## Conclusions

This study showed that SNP rs10741657 in the CYP2R1 gene was associated with the risk of NSCLC death, especially in elderly NSCLC patients, and not being treated with chemotherapy. Some of the VD pathway-related genetic polymorphisms may influence the prognosis of NSCLC.
